# Supplementary material for: High-Risk Human Papillomavirus E7 Alters Host DNA Methylome and Represses HLA-E Expression in Human Keratinocytes
Source: Sci Rep. 2017 Jun 16;7:3633. doi: 10.1038/s41598-017-03295-7 (PMC5473897; doi:10.1038/s41598-017-03295-7)

SUPPLEMENTARY INFORMATION

High-Risk Human Papillomavirus E7 Alters Host DNA Methylome and Represses *HLA-E* Expression in Human Keratinocytes

Louis Cicchini[a], Rachel Z. Blumhagen[b], Joseph A. Westrich[a], Mallory E. Meyers[a], Cody J. Warren[a], Charlotte Siska[b], David Raben[c], Katerina J. Kechris[b], Dohun Pyeon[a,d,]*

Department of Immunology and Microbiology, University of Colorado School of Medicine, Aurora, Colorado, USA[a]; Department of Biostatistics and Informatics, Colorado School of Public Health, Aurora, Colorado, USA[b]; Department of Radiation Oncology, University of Colorado School of Medicine, Aurora, Colorado, USA[c]; Department of Medicine, University of Colorado School of Medicine, Aurora, Colorado, USA[d]

*Address correspondence to dohun.pyeon@ucdenver.edu

Running Head: HPV E7 epigenetically represses HLA-E expression

SUPPLEMENTARY METHODS

Gene Expression Data Processing and Statistical Analysis. Data analysis was performed using R version 3.2.2 [1]. Gene expression data quality from the Affymetrix Human Genome U133 Plus 2.0 Array was evaluated using QC plots for RNA degradation and log density distribution for all probes for each sample. The data were filtered based on mas5 present/absent calls, where probesets were discarded if they had at least one absent call. After filtering, 27,146 probesets remained. Transcript expression for each probeset was normalized using RMA [2]. Two-sample Student's t-test was used to measure differential expression for each comparison where correction for multiple comparisons was performed using Benjamini-Hochberg False Discovery Rate (FDR) within each comparison. Differentially expressed probesets were identified based on absolute minimum change in expression of 30% and FDR < 0.05 [3]. Expression was visualized via heatmaps from normalized signal intensity data generated using Microsoft Excel [4].

Methylome Data Processing and Statistical Analysis. Methylation detection *p*-values were calculated for each probe on the Infinium HumanMethylation450 BeadChip Array where the methylated and unmethylated signals were compared to background signal using negative control probe intensities. Non-significant detection *p*-values greater than 0.05 were filtered out. After filtering, 485,531 probes remained. Subset-quantile within array normalization (SWAN) provided by the minfi package was performed for each sample [5,6]. For comparison with gene expression data, CpG probe annotation including nearby genes was determined using the minfi package. Differentially methylated positions (DMP) were determined by independent two-sample Student's *t*-test on the M values for each comparison where β values were reported for significant CpG sites. To correct for multiple comparisons, *p*-values were adjusted using FDR within each comparison [3] and CpG probes with FDR < 0.05 were determined significant. Identification of differentially methylated regions (DMR) was performed using the bumphunter method where CpG probes within 500 bp of each other are grouped together [7]. A permutation-

based method was used to determine the null distribution and assess significance. The proportion of permutations that had at least one region as extreme as the observed region is reported as the permutation p-value. For each comparison, regions meeting the 99th quantile of the smoothed area for the DMR from the permutation distribution and those having a permutation p-value < 0.05 were considered significant.

Bioinformatics. 100 bp of genomic sequence on both flanks of each DMR were fetched using the Galaxy platform (usegalaxy.org) [8–10] and enriched TF binding motifs were identified using MEME Suite [11]. Flanking regions were limited to 200 bp total due to MEME Suite processing constraints. Relative TF binding site enrichment was performed using the Analysis of Motif Enrichment (AME) tool with shuffled input sequences as control DNA. The UCSC Genome Browser (hg19) was used to assess DNase I hypersensitivity (from ENCODE V3 using 125 cell types), H3K27Ac (ENCODE using 7 cell types), and transcribed human RNAs (GenBank) near the *HLA-E* CGI. miRNA Target Prediction and Functional Study Database (miRDB, mirdb.org) analysis mining was performed using default settings. Pathway analysis was performed using Reactome (reactome.org). Submitted gene lists were restricted by FDR < 0.05 and magnitude 30% change in expression using NIKS expression as baseline.

Quantitative Reverse Transcription-PCR (RT-qPCR). Total RNA was extracted from NIKS, NIKS-16, NIKS-18, and NIKS-16ΔE7 cells using High Pure RNA Isolation Kit (Roche Applied Science) and cDNA was prepared using the Transcriptor First Strand cDNA Synthesis Kit (Roche Applied Science) according to the manufacturer’s instructions. Real-time PCR was performed with specific primers (**Supplementary Table S7**) using the Bio-Rad CFT Connect Real-time System and FastStart Universal SYBR Green Master (Roche Applied Science). Data were normalized by the level of β-actin mRNA.

Flow Cytometry. A series of normal keratinocytes were dissociated to single cell populations by incubating in citric saline buffer (135 mM potassium chloride, 15 mM sodium citrate) to preserve cell surface proteins[12]. Cells were fixed in 4% paraformaldehyde, permeabilized with 0.1% saponin, and incubated overnight with anti-human HLA-E (PE conjugate, clone 3D12) or anti-human HLA-B/C (APC conjugate, clone B1.23.2) antibodies (Thermo Fisher Scientific). The stained cells were filtered through 35 µm cell strainer (Corning Life Sciences) immediately before analysis on an LSRII flow cytometer (Becton Dickinson) using FACSDiva collection software. Data were analyzed using FlowJo software.

REFERENCES

1. R Development Core Team. R: a language and environment for statistical computing. *R Found. Stat. Comput.* **ISBN: 3-90,** (2011).
2. Irizarry, R. A. *et al.* Exploration, normalization, and summaries of high density oligonucleotide array probe level data. *Biostatistics* **4,** 249–64 (2003).
3. Benjamini, Y. & Hochberg, Y. Controlling the False Discovery Rate: A Practical and Powerful Approach to Multiple Testing. *J. R. Stat. Soc.* **57,** 289–300 (1995).
4. Metsalu, T. & Vilo, J. ClustVis: a web tool for visualizing clustering of multivariate data using Principal Component Analysis and heatmap. *Nucleic Acids Res.* **43,** W566-70 (2015).
5. Aryee, M. J. *et al.* Minfi: a flexible and comprehensive Bioconductor package for the analysis of Infinium DNA methylation microarrays. *Bioinformatics* **30,** 1363–9 (2014).
6. Maksimovic, J., Gordon, L. & Oshlack, A. SWAN: Subset-quantile within array normalization for illumina infinium HumanMethylation450 BeadChips. *Genome Biol.* **13,** R44 (2012).
7. Jaffe, A. E. *et al.* Bump hunting to identify differentially methylated regions in epigenetic epidemiology studies. *Int. J. Epidemiol.* **41,** 200–9 (2012).
8. Goecks, J., Nekrutenko, A., Taylor, J. & Galaxy Team. Galaxy: a comprehensive

approach for supporting accessible, reproducible, and transparent computational research in the life sciences. *Genome Biol.* **11,** R86 (2010).

9. Blankenberg, D. *et al.* Galaxy: a web-based genome analysis tool for experimentalists. *Curr. Protoc. Mol. Biol.* **Chapter 19,** Unit 19.10.1-21 (2010).
10. Giardine, B. *et al.* Galaxy: a platform for interactive large-scale genome analysis. *Genome Res.* **15,** 1451–5 (2005).
11. Bailey, T. L. & Elkan, C. Fitting a mixture model by expectation maximization to discover motifs in biopolymers. *Proc. Int. Conf. Intell. Syst. Mol. Biol.* **2,** 28–36 (1994).
12. Zhang, B. *et al.* Different methods of detaching adherent cells significantly affect the detection of TRAIL receptors. *Tumori* **98,** 800–803 (2012).

Supplementary Figure Legends

Supplementary Figure S1. Summary of biological pathways related to HPV16 E7-dependent gene expression changes. Pathways analysis was performed using Reactome (version 56, reactome.org) with genes specifically upregulated or downregulated (**Supplementary Table S1**) by HPV16 E7 described in **Fig 1b**. The global pathway diagrams shown are automatically generated by the Reactome database. Yellow nodes and lines indicate pathways and their associations significantly altered by HPV16 E7 expression. Pathway clusters showing significant E7-dependent changes are indicated with red bold text and specific biological pathways significantly changed (FDR-adjusted $p < 0.01$) are listed in **Supplementary Table S2**.

Supplementary Figure S2. Gene expression involved in matrix metalloproteinases, IL1 signaling, and antigen presentation is dysregulated by high-risk E7. Normalized gene expression levels in the pathways of (**a**) matrix metalloproteinases (*MMP1, MMP9, MMP10, MMP28*); (**b**) IL1 signaling (*IL1B, IL1RN, IL1R1, IL36G*) and (**c**) antigen presentation (*Sec31A, ITGAV, CTSL2, RANSEL*) are shown. (**d**) Expression of *IL1B, IL1R1, IL1RN* and *IL36G* was validated by RT-qPCR with total RNA extracted from NIKS, NIKS-16, NIKS-18, and NIKS-16ΔE7 cells and normalized by β-actin expression. *P*-values were calculated by Student's *t* test and indicated on the graphs (**a-c**) or by asterisks as follows (**d**): $*p < 0.0001$, $**p < 0.0005$, $***p < 0.005$, $****p < 0.05$.

Supplementary Figure S3. Regulatory elements and non-coding RNAs in the *HLA-E* CGI. Regulatory elements were predicted near the *HLA-E* CGI in chromosome 6 (Chr:6) using the UCSC Genome Browser (version hg19, genome.ucsc.edu). The *HLA-E* CGI is shown as a green bar. The locations of ncRNAs identified by RNA sequencing, DNase I hypersensitivity, and transcription factor binding sites identified by ChIP-seq (EZH2, ELF1, UBTF, CCNT2, GABPA,

E2F6, MAX, HMGN3 and ELF1) are indicated as black bars. The locations of histone H3 lysine 27 acetylation (H3K27Ac) are shown with different colors indicating different cell types (GM12878, H1-hESC, HSMM, HUVEC, K562, NHEK, NHLF) in which acetylation was observed.

Supplementary Figure S4. Gating of flow cytometry. Representative flow cytometry parental gating strategy for NIKS cells, and derivatives, by side (SSC-A) and forward (FSC-A) scatter prior to evaluating HLA expression. Groups include untreated keratinocytes described in **Fig 6a-f**, as well as mock and 5-aza treated keratinocytes described in **Fig 6g-h.**

Supplementary Figure S5. Controls for flow cytometry. Unstained, HLA-E, and HLA-B/C single stained NIKS cells were analyzed by flow cytometry to evaluate staining specificity and ensure no spectral overlap by the fluorophores. Histograms from each group are shown in both HLA-E and HLA-B/C channels. The overlay of the histograms demonstrates that there is no spectral overlap between the fluorophores, and distinct positive populations are observed in the respective channels.

Supplementary Figure S6. E7 expression in NIKS cells. E7 gene expression was validated by RT-PCR using specific primers (**Supplementary Table S7**).

Supplementary Figure S7. Reduction of HLA-E DNA methylation by 5-aza treatment. MSP was performed with gDNA from NIKS-16 cells mock treated (mock) and treated with 5-aza (5-aza) using specific primers (**Supplementary Table S7**).

Figure S1

a

E7-dependent Gene Upregulation

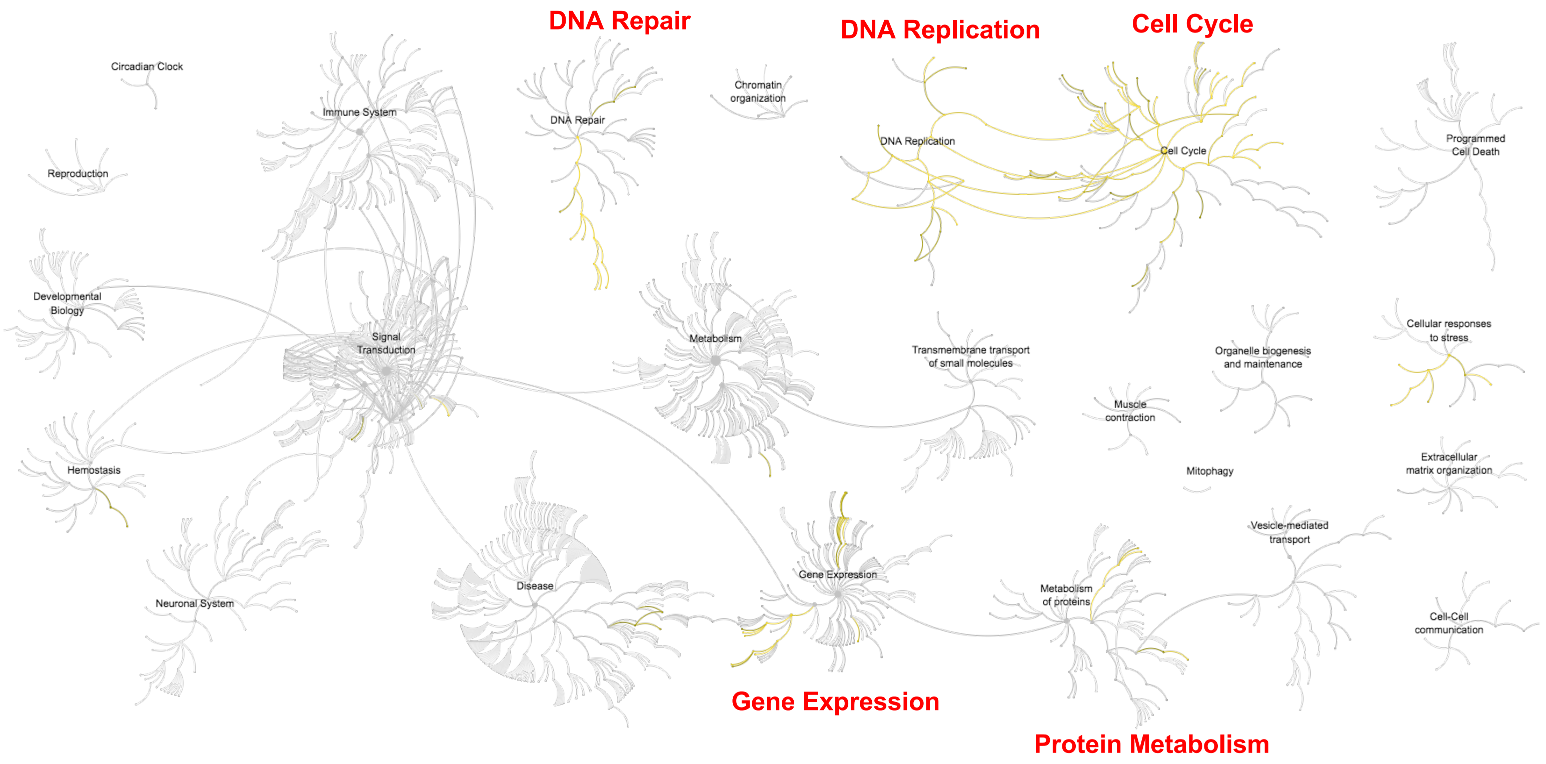

b

E7-dependent Gene Downregulation

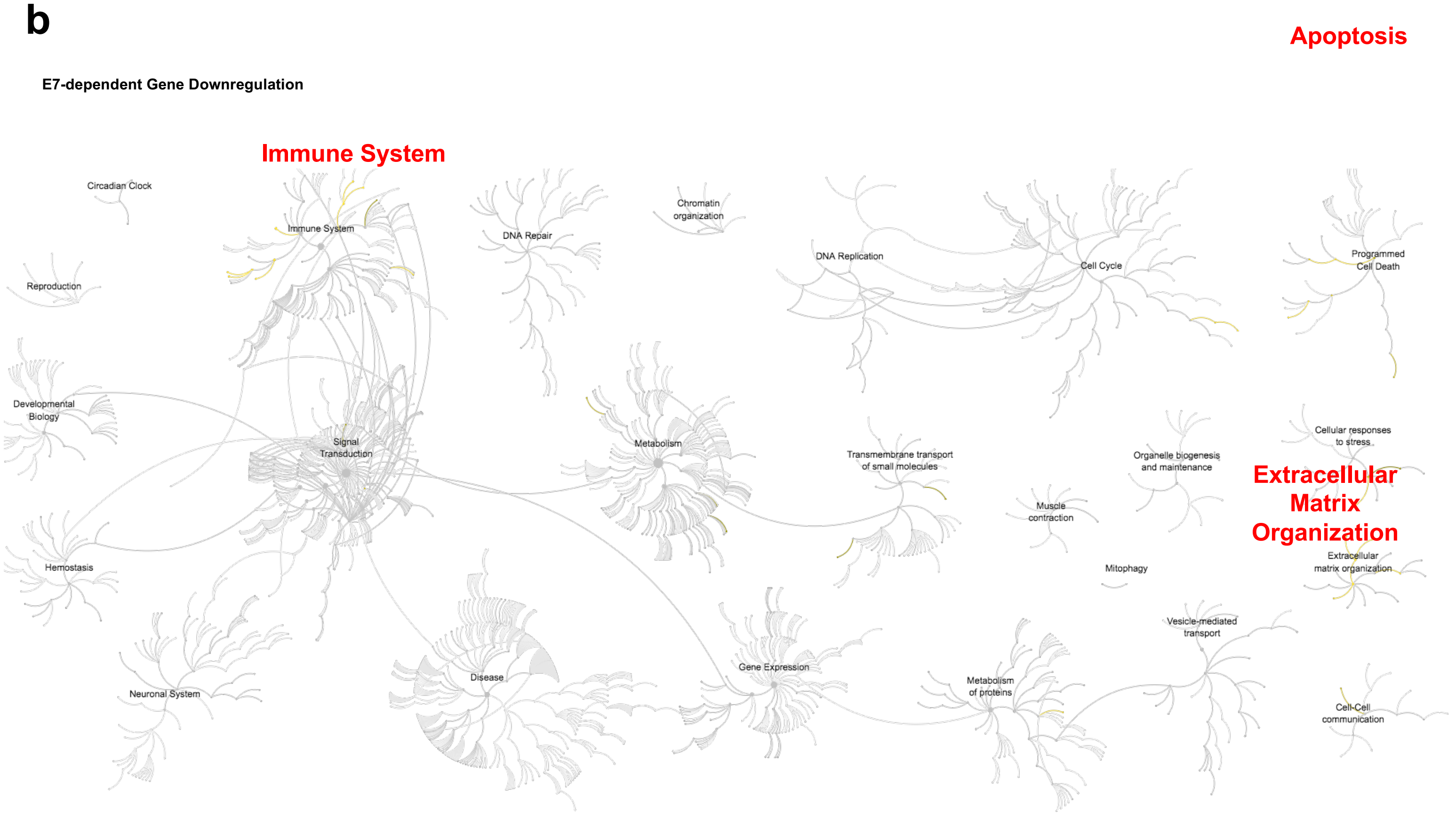

Figure S2

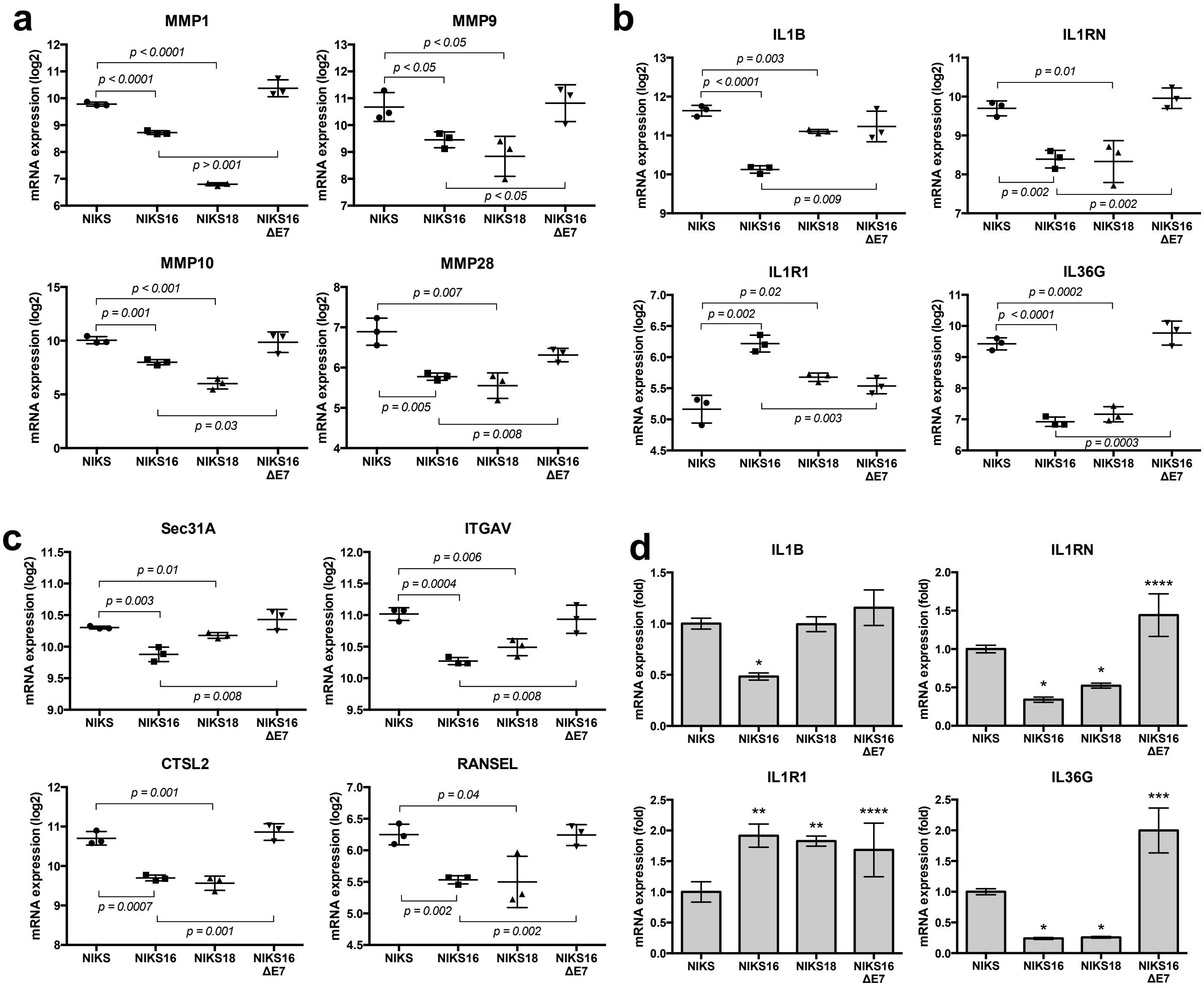

a
MMP1
MMP9
MMP10
MMP28
b
IL1B
IL1RN
IL1R1
IL36G
c
Sec31A
ITGAV
CTSL2
RANSEL
d
IL1B
IL1RN
IL1R1
IL36G
mRNA expression (log2)
mRNA expression (fold)
NIKS
NIKS16
NIKS18
NIKS16 ΔE7

Figure S3

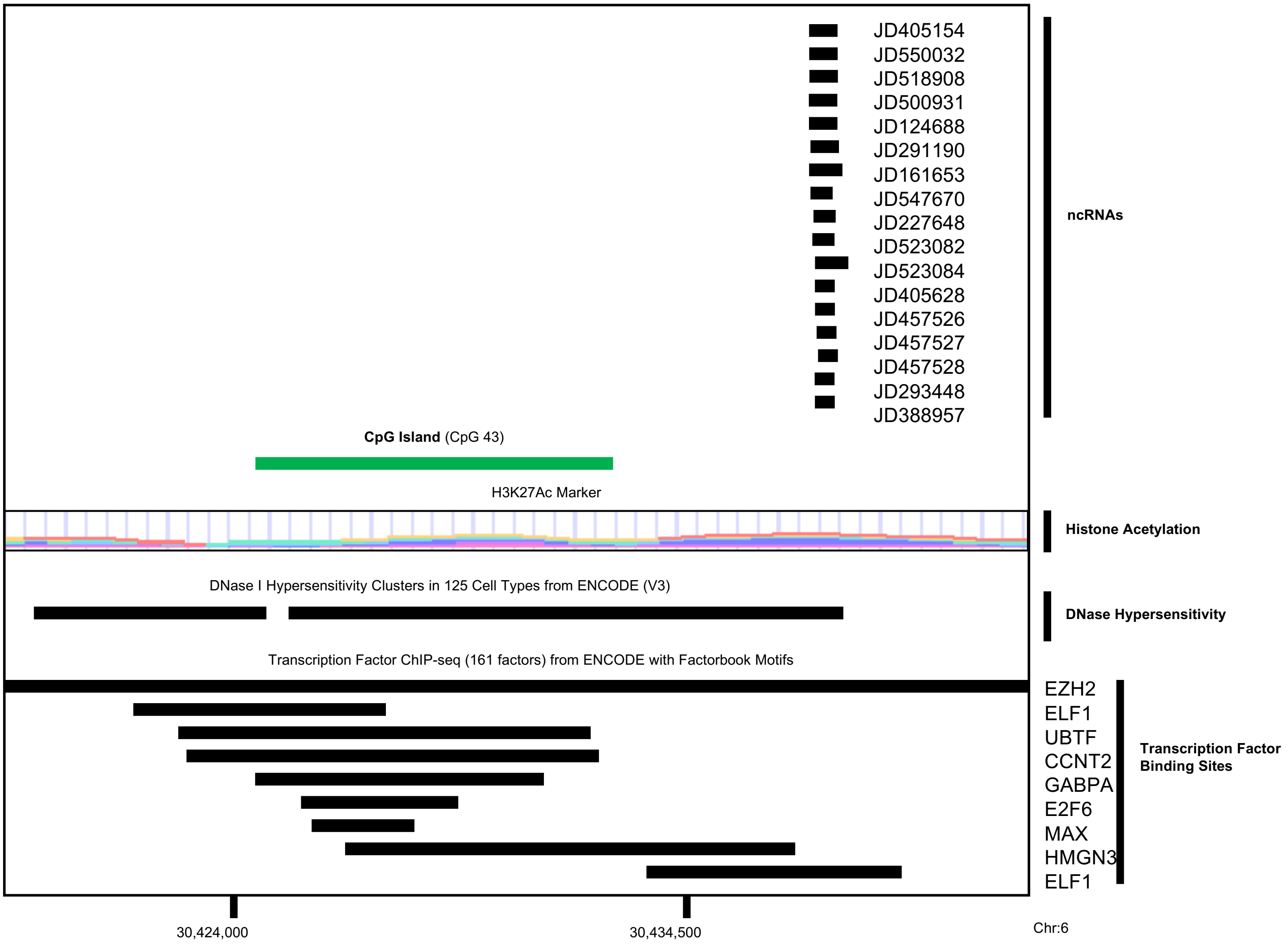
JD405154
JD550032
JD518908
JD500931
JD124688
JD291190
JD161653
JD547670
JD227648
JD523082
JD523084
JD405628
JD457526
JD457527
JD457528
JD293448
JD388957
ncRNAs
CpG Island (CpG 43)
H3K27Ac Marker
Histone Acetylation
DNase I Hypersensitivity Clusters in 125 Cell Types from ENCODE (V3)
DNase Hypersensitivity
Transcription Factor ChIP-seq (161 factors) from ENCODE with Factorbook Motifs
EZH2
ELF1
UBTF
CCNT2
GABPA
E2F6
MAX
HMGN3
ELF1
Transcription Factor Binding Sites
30,424,000
30,434,500
Chr:6

Figure S4

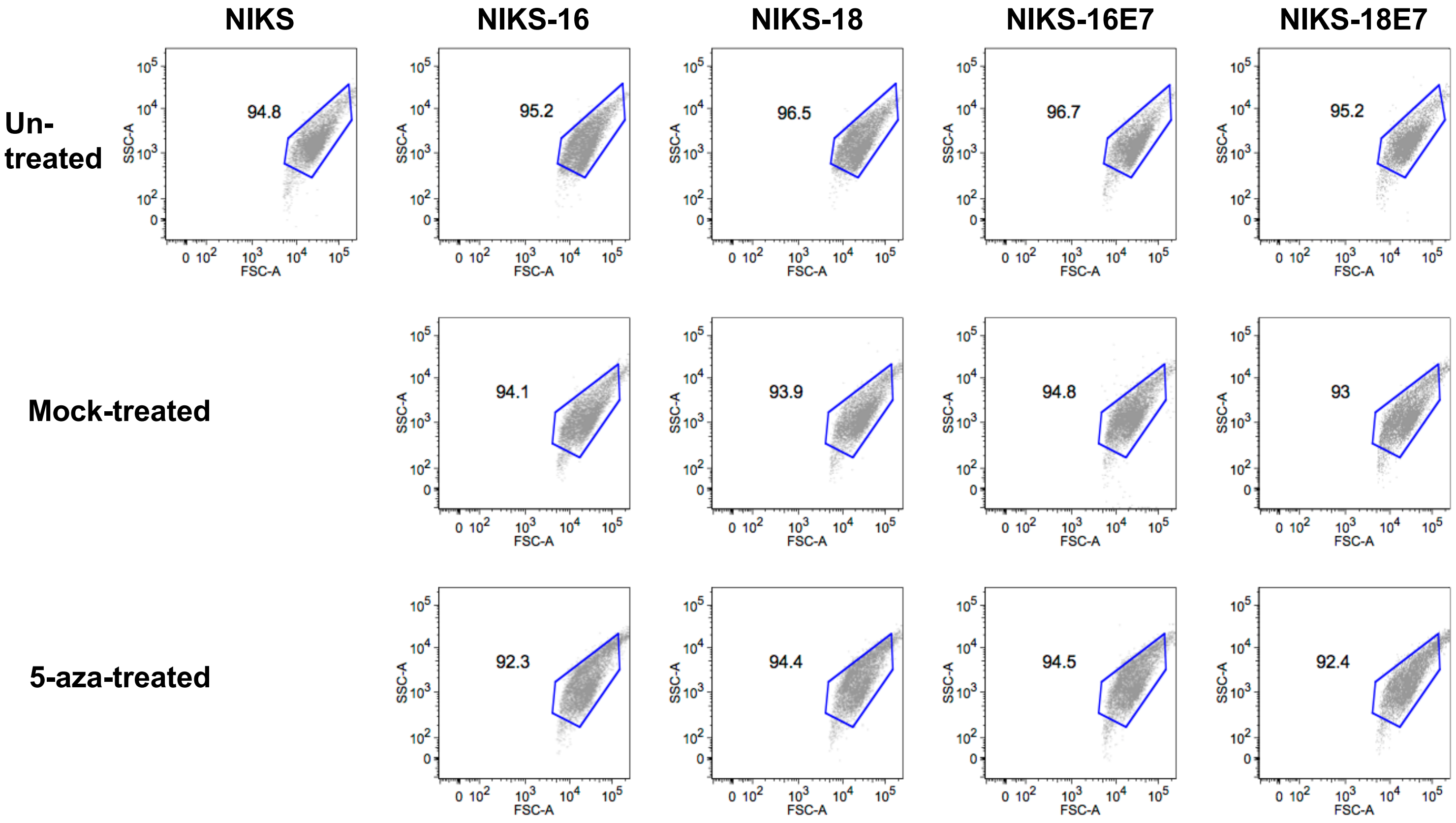

NIKS
NIKS-16
NIKS-18
NIKS-16E7
NIKS-18E7
Un-treated
Mock-treated
5-aza-treated
94.8
95.2
96.5
96.7
95.2
94.1
93.9
94.8
93
92.3
94.4
94.5
92.4
SSC-A
FSC-A

Figure S5

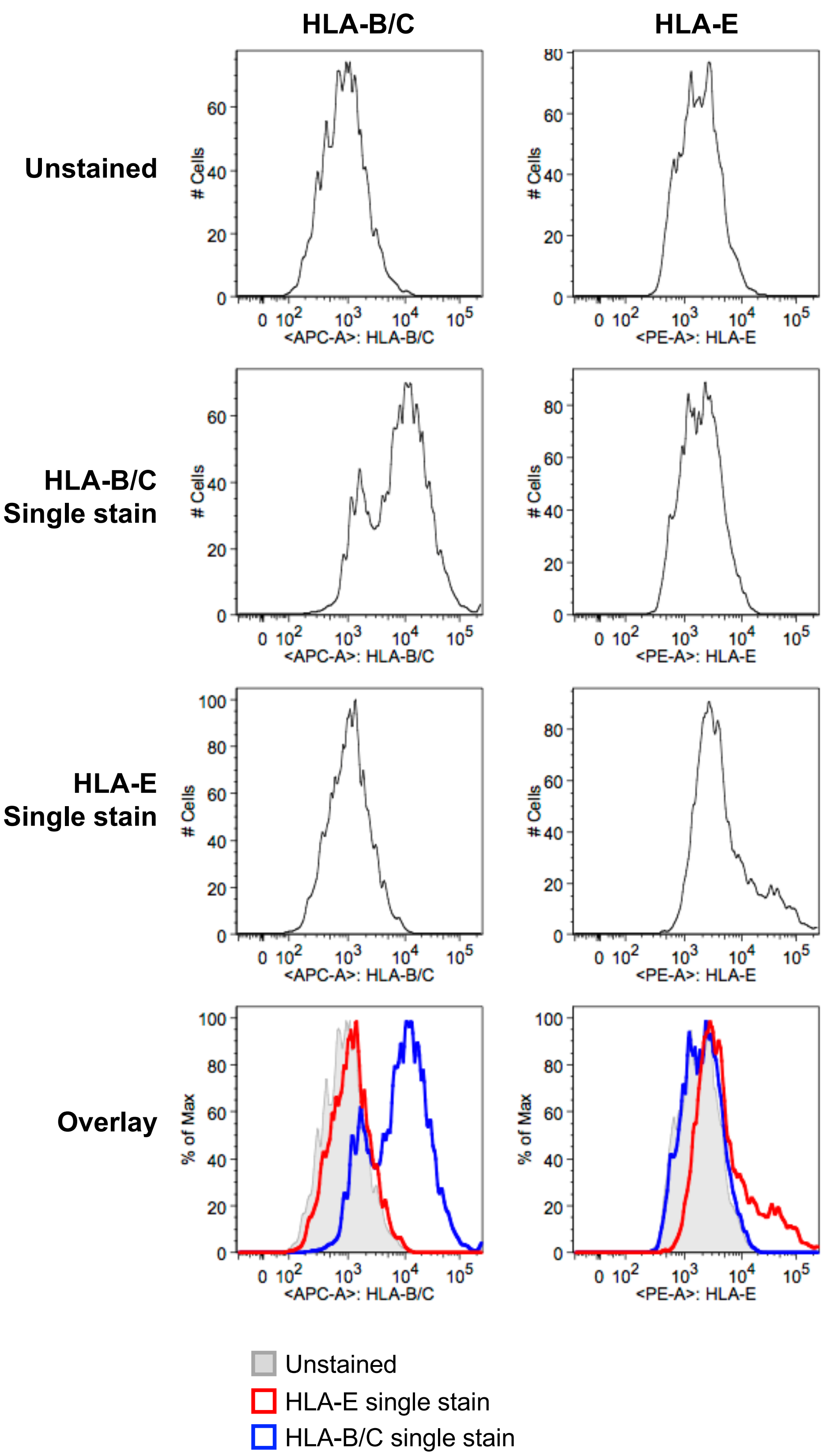

HLA-B/C
HLA-E
Unstained
HLA-B/C
Single stain
HLA-E
Single stain
Overlay
Cells
% of Max
<APC-A>: HLA-B/C
<PE-A>: HLA-E
Unstained
HLA-E single stain
HLA-B/C single stain

Figure S6

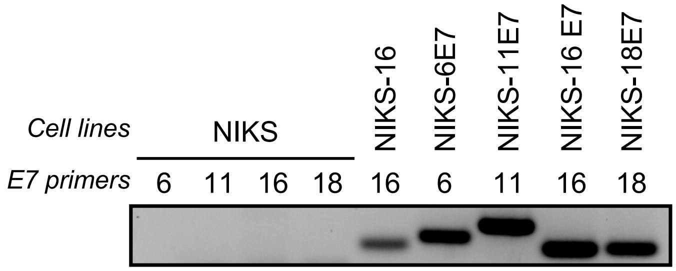

Figure S7

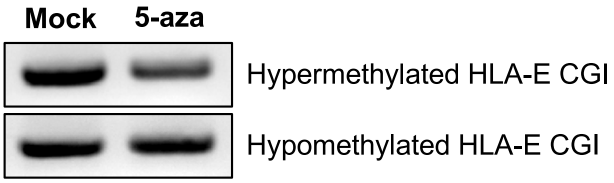

Supplement: Supplementary file 1 — Supplementary Information [file 41598_2017_3295_MOESM1_ESM.pdf]
